# Use of a Mixture Statistical Model in Studying Malaria Vectors Density

**DOI:** 10.1371/journal.pone.0050452

**Published:** 2012-11-21

**Authors:** Olayidé Boussari, Nicolas Moiroux, Jean Iwaz, Armel Djènontin, Sahabi Bio-Bangana, Vincent Corbel, Noël Fonton, René Ecochard

**Affiliations:** 1 International Chair in Mathematical Physics and Applications, Université d'Abomey-Calavi, Abomey-Calavi, Bénin; 2 Service de Biostatistique, Hospices Civils de Lyon, Lyon, France; 3 Université de Lyon, Lyon, France; 4 Université Lyon 1, Villeurbanne, France; 5 Laboratoire de Biométrie et Biologie Evolutive, Centre National de la Recherche Scientifique – Unité Mixte de Recherche 5558, Villeurbanne, France; 6 Maladies Infectieuses et Vecteurs Écologie, Génétique, Évolution et Contrôle, Institut de Recherche pour le Développement, Montpellier, France; 7 Université Montpellier 1, Montpellier, France; 8 Université Montpellier 2, Montpellier, France; 9 Maladies Infectieuses et Vecteurs Écologie, Génétique, Évolution et Contrôle, Institut de Recherche pour le Développement, Cotonou, Bénin; 10 Centre de Recherche en Entomologie de Cotonou, Ministère de la Santé, Cotonou, Bénin; Centro de Pesquisas René Rachou, Brazil

## Abstract

Vector control is a major step in the process of malaria control and elimination. This requires vector counts and appropriate statistical analyses of these counts. However, vector counts are often overdispersed. A non-parametric mixture of Poisson model (NPMP) is proposed to allow for overdispersion and better describe vector distribution. Mosquito collections using the Human Landing Catches as well as collection of environmental and climatic data were carried out from January to December 2009 in 28 villages in Southern Benin. A NPMP regression model with “village” as random effect is used to test statistical correlations between malaria vectors density and environmental and climatic factors. Furthermore, the villages were ranked using the latent classes derived from the NPMP model. Based on this classification of the villages, the impacts of four vector control strategies implemented in the villages were compared. Vector counts were highly variable and overdispersed with important proportion of zeros (75%). The NPMP model had a good aptitude to predict the observed values and showed that: i) proximity to freshwater body, market gardening, and high levels of rain were associated with high vector density; ii) water conveyance, cattle breeding, vegetation index were associated with low vector density. The 28 villages could then be ranked according to the mean vector number as estimated by the random part of the model after adjustment on all covariates. The NPMP model made it possible to describe the distribution of the vector across the study area. The villages were ranked according to the mean vector density after taking into account the most important covariates. This study demonstrates the necessity and possibility of adapting methods of vector counting and sampling to each setting.

## Introduction

Malaria is still a major public health issue in Sub-Saharan Africa. In 2010, this region bore 91% of the global disease death burden estimated to 655,000 deaths [1]. Studying the risk of vector transmission is at the basis of every survey about the importance of malaria in a given zone. The overarching goal of vector control is to decrease the transmission of the malaria parasite *Plasmodium spp* to humans by mosquito vectors of the genus *Anopheles*. Among the recommendation of the World Health Organization (WHO) to fight malaria, the deployment of long-lasting insecticidal mosquito nets (LLIN) and indoor residual spraying (IRS) at national scale has shown important reductions of malaria burden although evidences of malaria resurgence have been recorded in several African countries [1], [2].

The most common indicator to evaluate vector control interventions such as LLIN and IRS relies on malaria transmission through estimation of the Entomological Inoculation Rate (EIR). EIR is the product of the Human Biting Rate (HBR; number of bites of malaria vectors per human per unit time) and the prevalence of *Plasmodium* infection in mosquitoes. HBR is usually measured using the Human Landing Catches (HLC) counting technique that is the method of reference to quantify the human-vector contact [3].

In 28 villages of Southern Benin, a recent cluster randomized controlled trial (RCT) aiming at comparing the efficacy of combined LLIN and carbamate IRS or carbamate-treated plastic sheeting (CTPS) with a background of LLIN coverage did not show benefits of the combination for reducing HBR and EIR [4]. In the study area, high variations in the density of malaria vectors were observed in time and space [5] and there were many localities with zero mosquitoes collected during several nights.

The most ancient and popular statistical distribution used to describe count data is the Poisson distribution that assumes equidispersion of the counts. However, in real datasets, these counts are often overdispersed [6]–[8] and there are various means to demonstrate it [9]–[11]. Among the causes of overdispersion is the excess of zeros. Within the context of malaria vectors counts, the excess of zeros may result from the absence of mosquitoes at some locations (houses, village…) or during some period of time (dry season, cold temperatures…).

To deal with such overdispersed data with excess zeros, Johnson and Kotz [12] introduced the zero-inflated Poisson model (ZIP); i.e., a Poisson mixture model that combines a point mass at zero with a Poisson count distribution. Later, Lambert [13] extended this model to allow for covariates. Another way to deal with count overdispersion is the use of the negative binomial (NB) model or, better, the zero-inflated negative binomial (ZINB) model constructed on the same principle as that of the ZIP.

Besides these well-known models, other finite mixture distribution models have been proposed (e.g., McLachlan and Peel [14]) and have been the object of numerous applications. In fact, these models extend the previous ones; instead of considering a mixture of two distributions as with the ZIP or the ZINB, they consider a mixture of three or more Poisson or NB distributions. In addition, a non-parametric approach of the maximum likelihood introduced by Aitkin [15] has shown to be an excellent tool to allow for overdispersion. An extension of this approach by the same author [16] allowed its application to repeated measurements. Thus, a non-parametric mixture of Poisson model (NPMP) seems adapted to take into account the frequent changes in vector counts in various sites of a study zone.

In the present work, we assessed the ability of Poisson, NB, ZIP, ZINB and NPMP to fit the distribution of counts of malaria vectors measured in 28 villages in southern Benin where a clinical trial was implemented to evaluate the efficacy of vector control interventions for malaria prevention [4]. Using a multivariate NPMP, we introduced a classification of the villages based on the mean vector density after adjustment for a set of environmental and climatic covariates. Then, we assessed the relationship between this classification and the vector interventions implemented in the villages. The results of this work will help design site-specific malaria vectors sampling.

## Methods

### Mosquito collection

The data analyzed in the present study stem from mosquito collections carried out every 6 weeks between January and December 2009 (i.e. 8 surveys) in 28 villages of the sanitary region of Ouidah-Kpomassè-Tori (OKT) in South Benin. Of the 58 villages screened at the baseline, 28 were enrolled. The other villages were excluded because they did not fulfill inclusion criteria i.e. distance between two villages >2 km, population size between 250 and 500 inhabitants with non-isolated habitations and absence of any local health care centre.

Entomological surveys were performed using the HLC technique, on two successive nights (22:00 to 06:00) at four sites (both indoor and outdoor) per village. Collectors were hourly rotated along collection sites and/or position (indoor/outdoor). Malaria vectors collected on humans were identified using morphological keys [17]. Only *Anopheles gambiae* and *Anopheles funestus* mosquito counts were considered in the present work because these are the main malaria vectors in West Africa [18]–[20] and practically the only present in the study area [5].

These villages were divided into four groups (seven villages per group) where four different vector control measures were implemented (see Corbel et al. [4] for details): i) targeted-coverage LLIN (TLLINs) destined to protect pregnant women and children <6 years old (the reference group); ii) universal-coverage LLIN destined to protect sleeping units (ULLINs), iii) TLLINs plus full IRS of carbamate every eight months (TLLIN+IRS), and ULLIN plus full CTPS taped to the upper part of the walls (ULLIN+CTPS).

### Ethics statement

The IRD (Institut de Recherche pour le Développement) Ethics Committee and the National Research Ethics Committee of Benin approved the study (CNPERS, reference number IRB00006860). The study was also registered with Current Controlled Trials, number ISRCTN07404145. All necessary permits were obtained for the described field studies. No mosquito collection was done without the approval of the village chief, the owner and occupants of the collection house. Mosquito collectors gave their written informed consent and were treated free of charge for malaria presumed illness throughout the study.

### Demographic, geographic and environmental data

The following data were collected: the average distance (in km) from each village to the nearest freshwater body (Toho lake), the presence of market gardening 2 km around each village, the presence of cattle farms inside the village, the presence of water conveyance in the village, and the population density. The layout (or structure) of each village was described by the distribution of its clusters of houses, these clusters being separated by vegetated strips. Two modalities were then considered: single-cluster vs. multi-cluster villages. Daily rainfall data from 8 weather stations were spatially interpolated to compute the cumulated rainfall (in mm) and the number of rainy days in each village during the 15 days preceding each survey. The Normalized Difference Vegetation Index (NDVI) was derived from a “Satellite pour l'Observation de la Terre (SPOT-5)” satellite image acquired on 12/28/2003. The mean NDVI was computed in a buffer area of 50 m diameter around each mosquito collection site (house).

### Checking overdispersion and excess of zero in the data

The mean-variance relationship regarding the number of collected malaria vectors was analyzed graphically to explore data dispersion. A linear relationship of slope 1 (variances equal to means) indicated a Poisson distribution without overdispersion whereas a linear relationship with slope >1 or a quadratic relationship indicated overdispersion. We also assess the “excess of zero” through a graphical representation of the distribution of vector counts.

### Approximation of the distribution of the data

The approximations of the distribution of the number of collected malaria vectors by the Poisson, ZIP, NB, ZINB and NPMP distributions were compared using the maximum likelihood (ML) estimation. Poisson, ZIP, NB and ZINB models were fitted using the function ***nlm*** [21], [22] in the ‘R’ software version 2.14.0. Parameters of the NPMP model were estimated with the function ***alldist*** [15], [23] which used an EM algorithm [24]. In this approach, the dispersion of the data is described by a probability law that does not take into account the hierarchical structure of the data. This is thus a “marginal” model. A graphical representation of comparison results is used to show the counts as well as the predictions given by each of the above-cited distributions.

### Multivariate Analysis

Given the hierarchical structure of the data collection system, another NPMP model was considered to allow for various components of the variance of the counts. In this model, the counts of malaria vectors were assessed according to environmental and climatic covariates with the “village” as a random effect. It is thus a “conditional” model (on “village”). The latter model allows for the following variables: average distance to Toho lake (in km), water conveyance (0 = absence, 1 = presence), market gardening (0 = absence, 1 = presence), cattle farms inside the village (0 = absence, 1 = presence), the layout of the village (0 = multi-cluster, 1 = single-cluster), population density (inhabitants per 100 m^2^), both the mean cumulated rainfall over the 8 surveys (in mm) and the deviation from this mean at each survey, both the mean cumulated number of rainy days over the 8 surveys and the deviation from this mean at each survey, both the averaged NDVI over the 4 collection houses per village and the deviation from this average for each house and, finally, the specific collection site (0 = inside of the house, 1 = outside of the house).

According to the current recommendation for the use of hierarchical models, each covariate was centered on its mean before introduction into the model [25]. Variable “survey” was introduced into the model as a fixed effect. Mosquito collections made inside or outside each house of each village were considered as repeated measurements within that village.

In the NPMP conditional model, the number of malaria vectors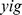 collected at a given site of a given village 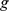 during a given night 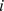 is supposed, conditionally to “the village”, to follow a mixture of four Poisson distribution. Each Poisson distribution has a mean 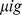 so that 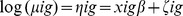. Note that 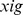 is the vector of values taken by the covariates, 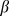 the corresponding fixed effects, 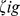 the random intercept specific to each village so that 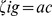, with probabilities 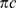 [25]. The values taken by 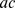 are called “latent variables”; *c* indicating each latent class, here fixed to four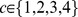. Hence, the density function of the model can be expressed as 
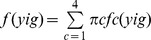
 where 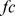 is the density function of a Poisson distribution with mean 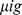. Thus, a non-parametric mixture model may also be called “latent class model”. The four values exp(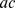) (one value for each latent class) are the predicted mean numbers of malaria vectors collected whenever all the model covariates, centered on their means, are equal to zero.

Function ***allvc*** [16], [23], a variant of ***alldist*** adapted for hierarchical data, was used for the latter model implementation in R software.

### Assessing the impact of vector control strategies

For each village, a posteriori probability of belonging to each class after adjustment on all the covariates is estimated by the NPMP conditional model. Here, “a posteriori probability” means the conditional probability for a village to belong to a given class, given the data. For a village 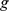 and a latent class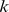, this probability can be expressed: 
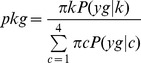
. In this expression, 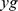
is the vector of observations in the village 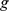 and 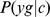 the probability of 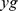 assuming membership in class 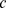.

Hence, each village is assigned to one of the classes based on the maximum of the a posteriori probabilities (MAP). This provides a classification of the villages according to the average number of malaria vectors collected at a given site over a given night after adjustment on all the covariates.

In order to assess the impact of TLLIN, ULLIN, ULLIN+CTPS and TLLIN+IRS vector control strategies, the village grouping for implementation of these vector control strategies and the classification resulting from the NPMP conditional model were compared using a Kruskall-Wallis test.

### About the number of the latent classes

The relevance of a NPMP model also called Poisson latent classes model, be it marginal or conditional, depends jointly on its ability to provide a close distribution to that of the observed counts and on its ability to assign each count one of the classes. Essentially, two criteria contributed to the choice of the number of classes: the closeness of the predicted values to the observed ones, which is the deviance expressed under the form of a Bayesian Information Criterion [26]; and, the ability of the model to assign each count one of the classes, which is expressed by the Entropy [27]. The Integrated Complete-data Likelihood (ICL-BIC) [28] is a combination of these two criteria; precisely, the BIC plus two times the entropy. Hence, the number of classes chosen for a latent class model is the one that maximizes the likelihood with low entropy equivalent to a minimum ICL-BIC.

## Results

### Entomological Data

**Figure 1:**
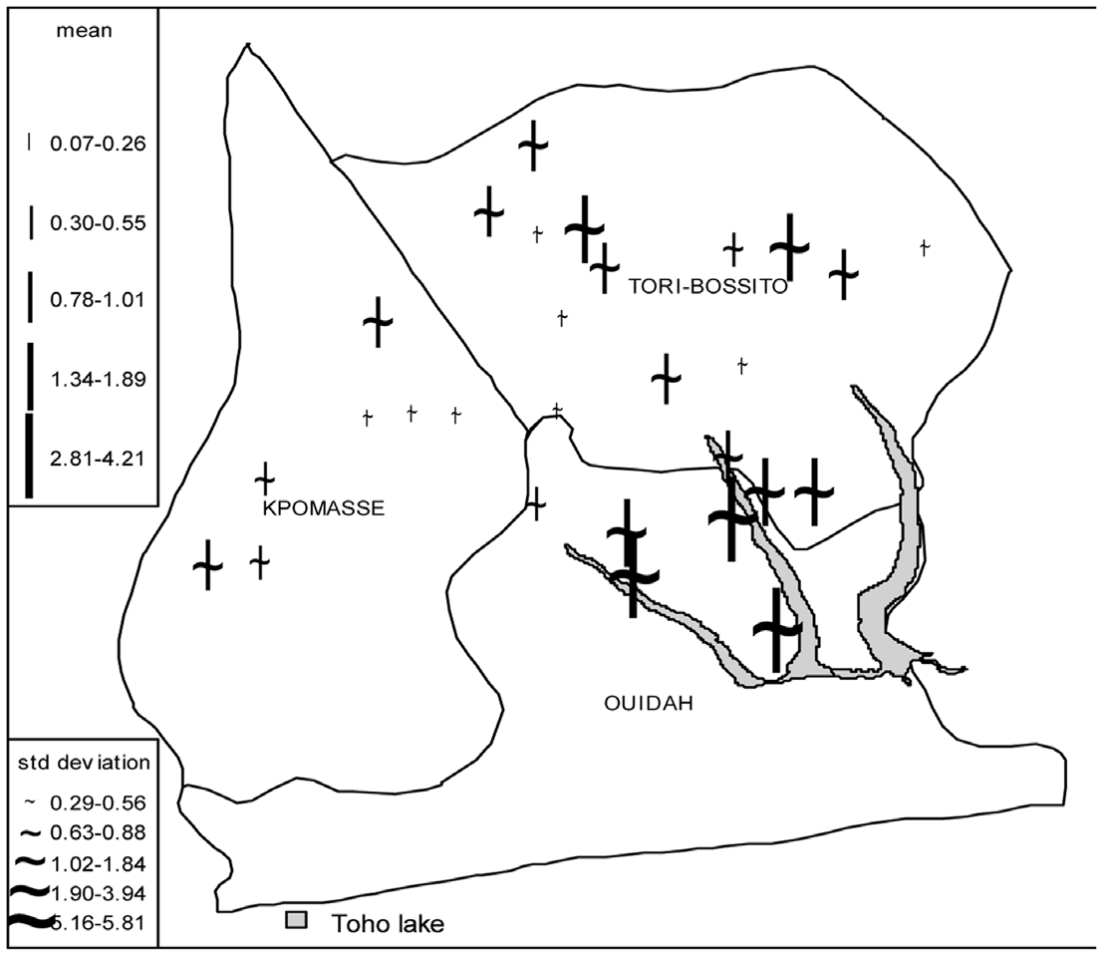
Means and standard deviations of the number of mosquitoes collected per site and per night at each of the 28 villages of the study.

Total of 2,994 malaria vectors were collected during 3,584 human-nights of mosquito collection. This corresponded to an average HBR of 0.835 bites per human per night. Among these vectors, 1,872 belonged to the *An. gambiae* complex and 1,122 belonged to the *funestus* Group. The density of anopheline collected changed over space and time (Figure 1). Indeed, the mean HBR in OKT ranged from 0.070 to 4.219 bites per human per night when the standard deviation ranged from 0.286 to 5.812. In most villages, high standard deviations corresponded to high means and between surveys the number of malaria vectors collected varied.

### Study of the dispersion

**Figure 2:**
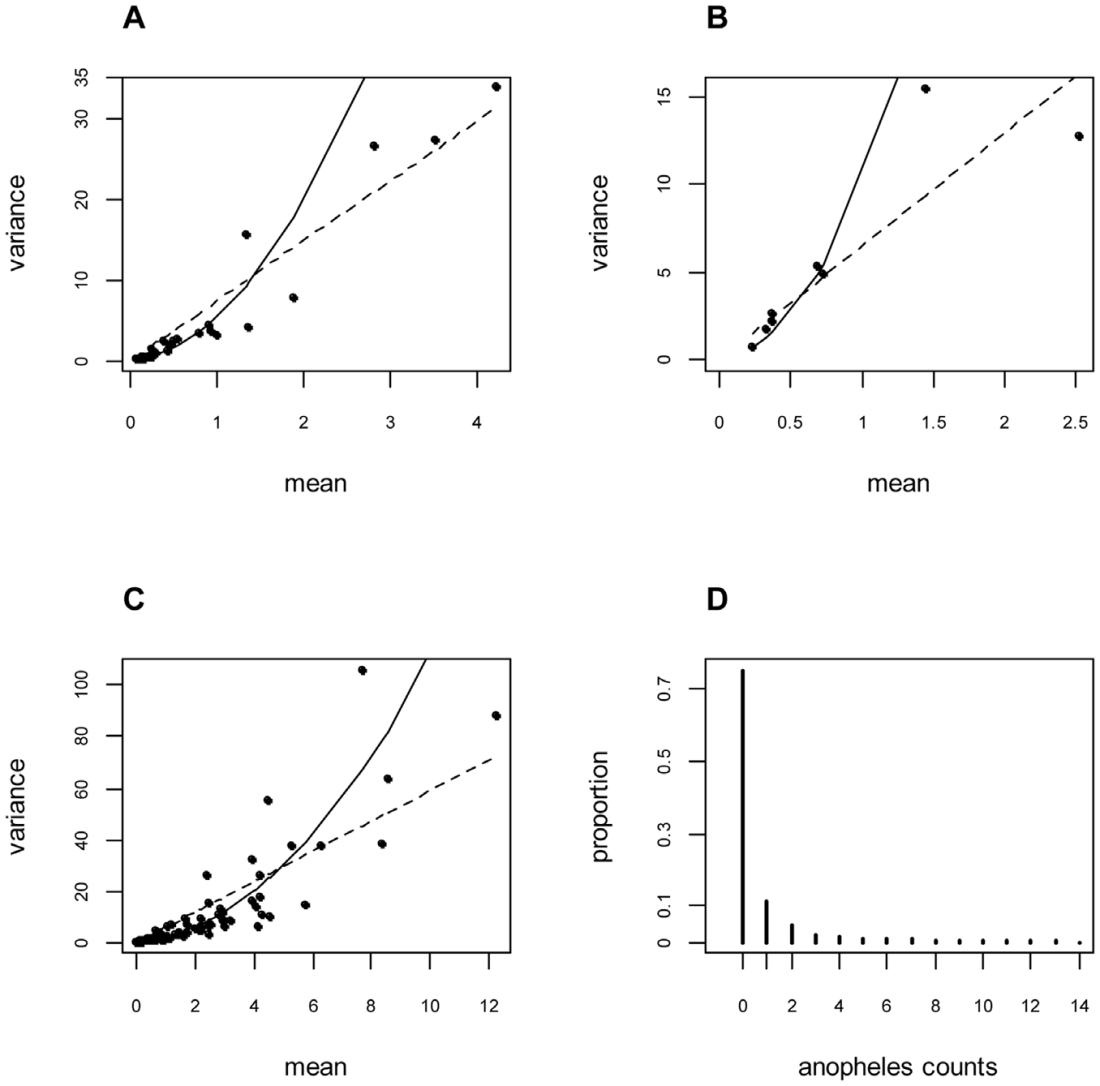
Mean-variance diagrams of the number of malaria vectors collected per village (Panel A), per mission (Panel B), and per village-mission (Panel C). Panel D shows a bar diagram of the distribution of mosquito counts at each collection site (the scale of the X-axis was limited to 14). On panels A, B and C: the dotted lines represent a linear link between the means and the variance (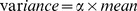 with 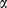 = 7.4, 6.48 and 5.9 respectively); the curves represent a quadratic link between mean and variance (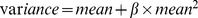 with 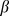 = 4.4, 8.9 and 1.1 respectively).

Village, survey, and village-survey mean numbers of malaria vector collected per night on humans were plotted with their corresponding variances in Figures 2A, 2B and 2C respectively. The assumption of mean-variance equality of the Poisson distribution was not met. Indeed, the variances were much higher than the means and the slopes of the linear relationships were >1 showing even quadratic relationships. This indicates overdispersion of the data.

### Distribution analysis

**Figure 3:**
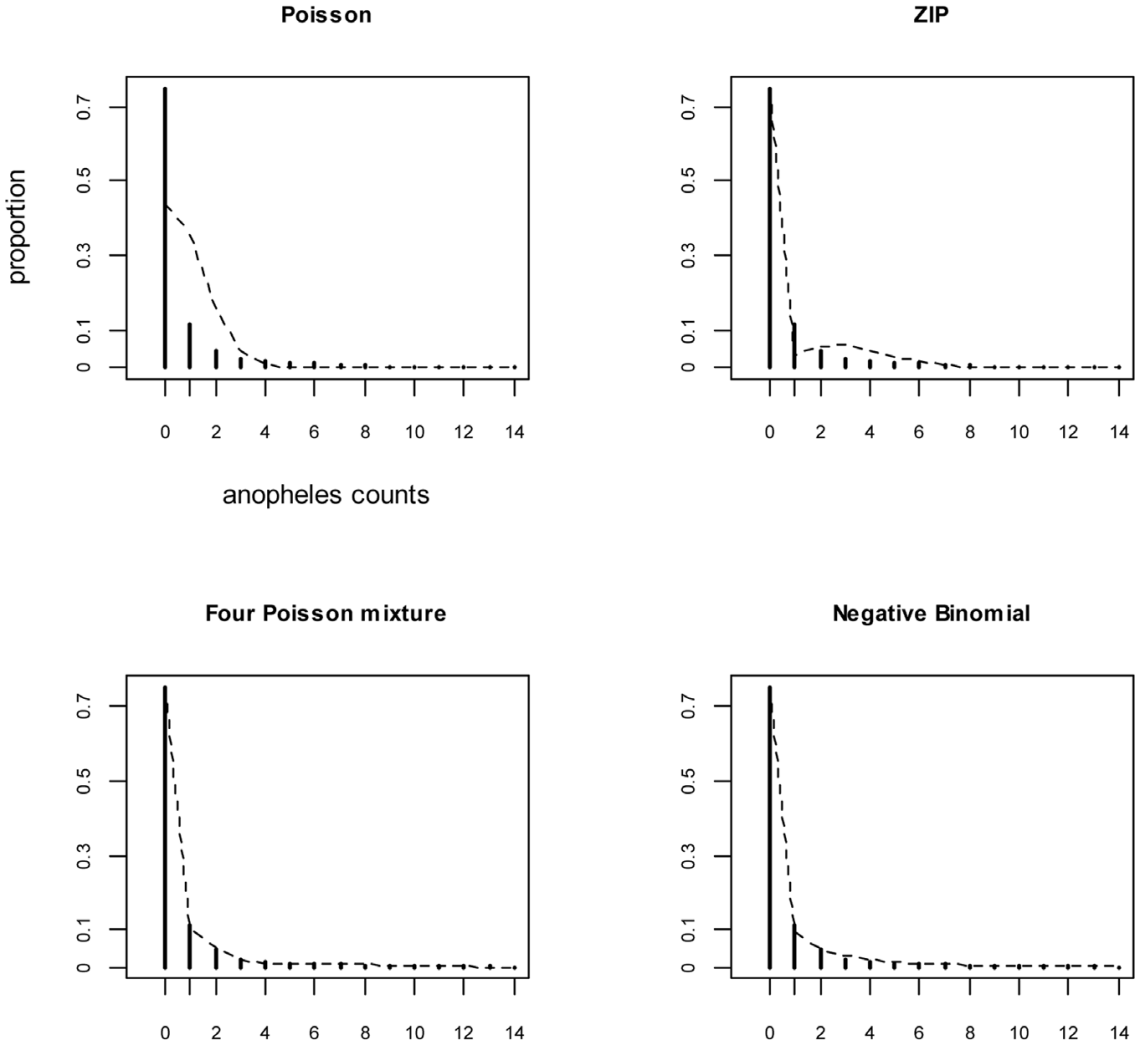
Observed and expected proportions of mosquito counts according to Poisson, ZIP, NPMP and NB distributions.

Frequency plot of the collected malaria vectors is shown in Figure 2D. The cases for which zero malaria vectors were collected represented 74.7% of the total. Table 1 shows the parameters for Poisson, ZIP, NPMP (the marginal model), NB and ZINB distributions. Based on the Poisson distribution with a mean of 0.835, we would expect 43.4% of zero (Figure 3A) which is significantly lower than that observed in the dataset. In contrast, ZIP, NB and NPMP models well predicted the proportion of zero (respectively 74.7%, 74.9%, and 74.7%; Figure 3). Excluding the sites where no anopheline were collected, the ZIP model estimated the mean number of collected anopheline per site per night at 3 but this does not solve the problem of data overdispersion. The NPMP model suggested that, in the study area, the sites where the anopheline counts over a single night would be generally high (mean 24.48) were rather rare (nearly 0.4%). However, the model estimated at 63% and 30%, respectively, the proportions of counts with 0 and 1 as the mean number of collected anopheline per night. The dispersion parameter estimated by the NB model was 0.156 indicating high overdispersion. Since the NB model allows for the excess of zeros, the proportion of zeros estimated by the ZINB model is nearly null. The NB and the ZINB models are therefore equivalent.

**Table 1:** Parameters and deviance as estimated by the Poisson, ZIP, NPMP, NB and ZINB models.

	Parameters			
Distribution	Mean (SE)	Proportion (SE)	Dispersion parameter	−2logL
Standard Poisson	0.835 (0.015)	1 (-)		13492.470
Zero-inflated Poisson (ZIP)				9229.370
*Zero-class*	0 (-)	0.736 (0.008)		
*Poisson*	3.169 (0.062)	0.264 (0.008)		
Poisson mixture model with 4 latent classes (NPMP)				7591.700
*Low*	0 (7×10^−6^)	0.630 (-)		
*Median-low*	0.923 (0.029)	0.296 (-)		
*Median-high*	6.555 (0.161)	0.070 (-)		
*High class*	24.480 (1.281)	0.004 (-)		
Negative Binomial (NB)	0.835 (0.038)	1 (-)	0.156 (0.007)	7581.856
Zero-inflated negative binomial (ZINB)				7581.856
*Zero-class*	0 (-)	3.6×10^−6^(1.7×10^−5^)		
*NB*	0.835 (0.038)	0.999 (1.7×10^−5^)	0.156 (0.007)	

−2logL: −2 times the log-likelihood

There were a significant decrease of the model deviance when a ZIP model was used instead of the standard Poisson model and also when the NPMP was used instead of the ZIP model (Table 1). The NB and ZINB fitted the data as well as the NPMP (deviance were not significantly different). Figure 3 shows the bar diagrams and the expected density probability curves of the counts with the Poisson, ZIP, NPMP and NB models. The curves relative to the NB and to the NPMP models are very similar and fit well the observed data distribution. Conversely, the curve relative to the standard Poisson model does not fit the observed frequency of counts between 0 and 10. Aside from the proportion of zeros, the ZIP model was not able to reproduce the observed proportions of counts ranging from 1 to 10.

### Choosing the number of latent classes

**Figure 4:**
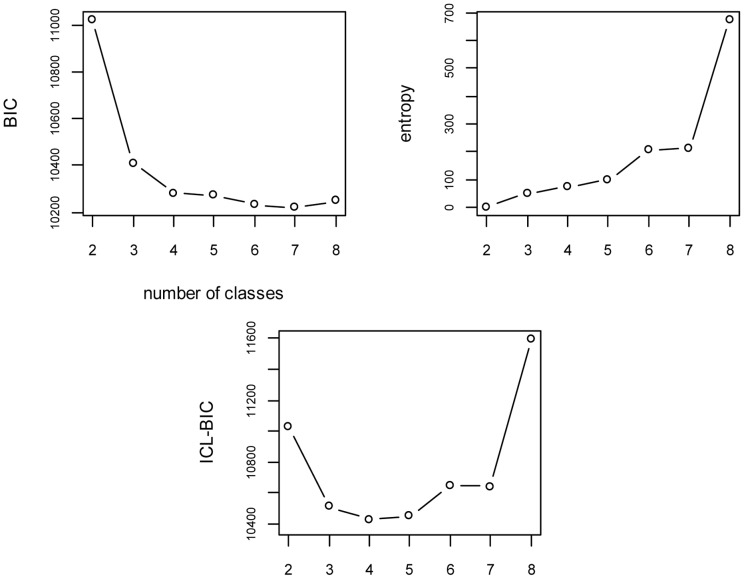
Changes in the values of the Bayesian Information Criterion (BIC), the entropy, and the Integrated Complete-data Likelihood (ICL-BIC) according to the number of latent classes.

Figure 4 shows for the NPMP model conditional on “village”, the progress of the BIC, the entropy, and the ICL-BIC according to the number of classes. Starting from 4 classes, the BIC became very low. The entropy augmented together with the number of classes. Their combination ICL-BIC was at its minimum with 4 classes. Therefore, the model with 4 latent classes was used to assess the number of malaria vector caught on humans according to climatic and environmental factors.

### Multivariate Poisson mixture analysis

Table 2 shows the relative risk of the fixed effects as estimated by the model. Presence of market gardening, population density, mean cumulated rainfall over the 8 surveys, mean cumulated number of rainy days over the 8 surveys and outdoor position were positively associated with the number of malaria vectors caught on human. On the other hand, distance to a freshwater body, presence of water conveyance, presence of cattle, single-cluster village houses, mean NDVI and deviation at each survey from mean cumulated number of rainy days over the 8 surveys were negatively associated with the number of malaria vectors caught on human.

**Table 2:** Estimations of the relationships between mosquito density and various geographical and environmental factors in OKT region according to the conditional NPMP model.

Level and covariate	Relative Risk (95% CI)
*Village*	
Distance to a freshwater body (per additional km)	0.885 (0.871–0.899)
Presence of water conveyance (Yes vs. No)	0.411 (0.348–0.485)
Presence of market gardening (Yes vs. No)	1.146 (1.016–1.292)
Presence of cattle (Yes vs. No)	0.817 (0.700–0.954)
Layout of the village (single- vs. multi-cluster)	0.466 (0.377–0.574)
Population density (per additional inhabitant/100 m^2^)	1.335 (1.079–1.651)
Mean rain quantity over all surveys (per additional mm)	1.325 (1.292–1.359)
Mean number of rainy days over all surveys (per additional day)	2.148 (1.675–2.754)
Mean NDVI (per additional grade)	0.849 (0.827–0.872)
*House*	
Deviation from the mean NDVI of the village (per additional grade)	0.990 (0.978–1.003)
Collection site (outside vs. inside)	1.182 (1.100–1.270)
*Mission*	
Deviation* from the mean rain quantity (per additional mm)	0.993 (0.989–0.997)
Deviation* from the mean number of rainy days (per additional day)	0.902 (0.827–0.984)

* Difference between the mean value over all surveys and the value at a given survey

Table 3 shows the random effects of the model. These are the predicted mean number of collected malaria vectors per night for each of the four latent classes when all the covariates are at their mean values. This table shows also the final classification of the villages according to their respective MAP. The mean number of malaria vectors collected ranged from 0.050 vectors per human per night in the 1st class (with only one village: Hekandji) to 0.713 in the 4th class (with 8 villages).

**Table 3:** Classification of the 28 villages according to the maximum a posteriori probability (MAP) of belonging to each class after adjustment on all other covariates.

Village	Latent class	Mean number of mosquitoes	Proportionof villages	MAP
Hekandji	1	0.050	0.036	0.992
Aidjedo	2	0.137	0.218	0.997
Assogbenou				1
Ayidohoue				0.990
Dokanmey				0.998
Hounkponouhoue				1
Abenihoue				1
Adjame	3	0.324	0.466	1
Amoulehoue				1
Adjahassa				0.924
Kindjitokpa				1
Vidjinnagnimon				1
Guezohoue				1
Hla				1
Agokon				0.968
Dekponhoue				0.998
Lokohoue				1
Todo				1
Wanho				1
Zoume				0.994
Agouako	4	0.713	0.280	0.775
Hinmadou				0.925
Manguevie				0.925
Satre				0.925
Soko				0.925
Tanto				0.925
Tokoli				0.925
Agadon				0.925

A Kruskal-Wallis test did not show a significant association between villages classification obtained from the model and the villages grouping for vector control strategies (Chi2 = 2.029, p-value = 0.566). Thus, according to HBR, a significant difference in term of impact of vector control strategies (TLLIN, ULLIN, ULLIN+CTPS and TLLIN+IRS) is not showed.

## Discussion

Knowledge of malaria vector density in a given area is often needed for implementing and evaluating vector control interventions. This requires vector counts at several sites of the area and statistical analyses of these counts.

McCullagh and Nelder [29] asserted that whenever the variable of interest is a count, its distribution is often an overdispersed Poisson distribution. The present data are another illustration of this assertion. The first part of this work aimed at comparing which distribution among Poisson, NB, ZIP, ZINB and NPMP better fit on counts of malaria vectors recorded using the HLC technique. Both NB and NPMP models dealt with the excess of zero, with overdispersion and provided the best predictions of the distribution of the observed data. However, unlike NB model, the NPMP does not do any further assumption about the distribution of the means of malaria vectors counts. Besides, the hierarchical structure of the observed data was taken into account by a NPMP model conditional on “village”. Based on a posterior probability criterion, the NPMP model allowed ranking the villages in four latent classes according to the mean of vector density after adjustment on environmental and climatic covariates. The optimal number of latent classes was established on conventional criteria. Furthermore, the part each covariate played in the variability of malaria vector density in the area was estimated by the fixed effects of the model. However, the present study could not take into account all the possible hierarchical levels of the data because of the limits of software R in dealing with latent classes. Indeed, function ***allvc*** of package “npmlreg” cannot deal with more than two levels. We considered thus the catches at all sites of the same village as repeated measurements of the same variable. Therefore, we were not able to take into account the possible correlation between the counts from houses within the same village [30]. “Human bait” is another level that could induce correlation in the data but there is no sufficient information about all mosquito collectors. Besides, the rotation of the collectors during data collection reduces considerably such a correlation. “Season” could be another possible level of correlation; it was taken into account through rainfall data which is the main seasonal factor in the context of malaria vector density.

Moreover, the numbers of collected vectors during the 8 surveys are assumed to be uncorrelated although one may speculate about a correlation structure along time. Nevertheless, the correlation between mosquito counts from successive surveys is deemed to be very low because the time span between two successive surveys is 6 weeks whereas the lifespan of the vectors is only 3 to 4 weeks. Studying the correlation between counts from two nights during the same survey may reveal interesting results.

In southern Benin, both spatial and temporal heterogeneities in vector densities were mentioned by Djènontin et al. [5]. This can be explained by some factors we found associated with the density of malaria vectors. Firstly, cumulated rainfalls during the 15 days preceding the catches were positively associated with vector density as previously reported in Benin [30]. Moreover, the mean number (over all surveys) of rainy days was positively associated with the vector density whereas the deviation at each survey from this mean was negatively associated with the vector density. This suggested that high frequency of rainy events might flush out vectors breeding sites [31]. The vector density was lower in villages with water supply; this could be due to the absence of water storages that could have provided breeding sites for malaria vectors [32], [33]. Moreover, the presence of irrigated market gardening could have provided breeding sites [34], [35] and then, increased the density of vectors in villages closed to this activity as previously observed in Benin [36]. Permanent freshwaters of the Toho Lake could also have provided breeding sites for both *An. funestus* and *An. gambiae* [37]–[39] that are both present in our study area [5]. This explains why the vector density decreased when moving away from freshwater bodies as showed by Amek et al. [40] in Western Kenya. The presence of cattle was negatively correlated with vector density suggested that a part of the vector population could have bite on cattle instead of human. More vectors were caught in multi-cluster villages than in single-cluster villages. This might indicate that a multi-cluster village layout might increase the attractiveness of the village for malaria vectors because of the extra vegetation surrounding houses. Thus, the attractiveness of a multi-cluster village may be higher than that of a single-cluster village of same size. Catches were also more abundant outside than inside the houses. This indicates an exophagic behavior of malaria vectors in the study area. As suggested by two studies in the OKT region [4], [41], a part of the exophagic population of vectors could have avoided indoor residual insecticides.

One unexpected finding of the present study was that the NDVI was negatively correlated with the density of malaria vectors. This finding contrasts with several studies that used satellite imagery at a lower resolution [42], [43] but agrees with a study carried out in Burkina Faso that used the same SPOT images than ours [44]. In this study, the authors found a negative relationship between the larval productivity in ponds and the NDVI calculated from high resolution SPOT images. Indeed, a high NDVI might reflect the presence of submerged vegetation or water covered with vegetation that are usually related to very high *Anopheles* larval densities [44]–[46]. Moreover, the NDVI usually decreases with freshwater and unvegetated surfaces likely to provide breeding sites for the malaria vectors [37], [47]. Nevertheless, the discussion about the NDVI effect can be more complex because of the co-existence in the region of two major malaria vectors with different breeding-site requirements.

In this work, villages were ranked into four classes of increasing mean malaria vector density but we were not able to find any relationship between this grouping structure and the vector control intervention implemented in the village. This confirms the finding of Corbel et al. [4] who demonstrated with the same data, that vector density was not significantly different between treatment arms (TLLIN, ULLIN, TLLIN+IRS, and ULLIN+CTPS).

In conclusion, we found that the NPMP model was useful to assess the relationships between vectors density and villages or environmental characteristics. It might therefore be an efficient tool to compute risk maps of the host-vector contact. Moreover, the NPMP model provided a classification of the villages after taking into account some covariates. Such a classification could be used at a pre-study step to improve the study design of mosquito collection and adapt the sampling effort according to the village characteristics, especially in region with high spatial and temporal heterogeneities of mosquito density, like in the OKT region. Furthermore, NPMP model could help in the study design of RCT when a stratified sampling is needed. The same model may be adapted and used in other settings for the study of the distribution of vectors of other diseases.
